# Transhiatal versus transthoracic esophagectomy for esophageal SCC: outcomes and complications

**DOI:** 10.1186/s13019-022-01912-9

**Published:** 2022-06-09

**Authors:** Ehsan Soltani, Habibollah Mahmoodzadeh, Azadeh Jabbari Nooghabi, Mehdi Jabbari Nooghabi, Khosrow Ravankhah Moghaddam, Ehsan Hassanzadeh Haddad

**Affiliations:** 1 Surgical Oncology Research Center, Mashhad University of Medical Sciences, Mashhad, Iran; 2 Cancer Institute, Tehran University of Medical Sciences, Tehran, Iran; 3 Department of Statistics, Ferdowsi University of Mashhad, Mashhad, Iran

**Keywords:** Esophagectomy, Transthoracic, Transhiatal, Complication

## Abstract

**Background:**

Transhiatal esophagectomy (THE) and transthoracic esophagectomy (TTE) are both accepted procedures for esophageal cancer but still the most effective surgical approach continues to be controversial. This study aimed to determine post-operative complications and outcomes of TTE compared with THE.

**Methods:**

A retrospective analysis was performed on data of 243 adult patients with resectable esophageal cancer who underwent THE or TTE between December 2016 and October 2018. Demographic data, consisting of preoperative co-morbidities, disease stage, and perioperative morbidity and mortality were collected.

**Results:**

Among the patients, 99 individuals (40.7%) had a transhiatal resection and 144 (59.3%) had a transthoracic resection. Most patients (83.1%) were above 50 years old with no significant difference between groups (*p* = 0.297). The frequency distribution of comorbidities was similar in both groups. The most common site of the tumor in TTE group was middle esophagus and in THE group was lower esophagus. The most common complication was recurrence of dysphagia which was more common in THE group without significant difference. The other complications including pulmonary and cardiac events, tracheal and recurrent laryngeal nerve injury, chylothorax and anastomosis stricture did not differ between the groups. The operative mortality within 30 days after the operation was 2.8% with significant difference favored the THE group (THE 0%, TTE 5.2%, *p* = 0.033).

**Conclusion:**

Because of the controversies, the decision on the type of surgical technique in esophageal cancer treatment hinges on patient’s co-morbidities, cancer stage, tumor location and surgeon’s experience.

## Introduction

Unfortunately, esophageal cancer is a fatal disease with a high rate of local and distant recurrence and poor survival. The overall 5-year survival rate is about 12–28% and based on some studies above 50% of patients may present with unresectable or metastatic disease, however in the Middle East Report, the rate of unresectability and overall survival are more promising [1–4]. In the early stage of esophageal cancer, the mainstay of treatment is esophagectomy [5]. It is noticeable that a large number of patients are in age range of the elderly, usually with several co-morbidities, which can influence the operative results and conduce high rate of hospital mortality (about 6% in some centers) [1, 6, 7]. Although transthoracic three fields (TTE) and transhiatal two fields (THE) esophagectomy are the most popular operation techniques, the best surgical approach is still controversial. The purpose of this study was to compare post-operative complications and outcomes of transhiatal and transthoracic esophagectomy.

## Materials and methods

This retrospective study enrolled 243 patients with thoracic esophageal cancer from December 2016 to October 2018 after obtaining ethics committee approval (Mashhad University of Medical Sciences, Iran, No. 931219). Patients’ characteristics are presented in Table 1. The study participants underwent elective esophagectomy for primary esophageal cancer in Ghaem and Taleghani hospitals (Mashhad University of Medical Sciences, Iran) and Cancer Institute (Tehran University of Medical Sciences, Iran).

**Table 1:** Demographic variables of the patients

Group	TTE	THE	Total	*p* value
Frequency of group	144 (59.3%)	99 (40.7%)	243	
*Sex*				
Female	66 (45.8%)	60 (60.6%)	126 (51.9%)	0.027
Male	78 (54.2%)	39 (39.4%)	117 (48.1%)	
*Age*				
18–50	21 (14.7%)	20 (20.2%)	41 (16.9%)	0.297
50–85	122 (85.3%)	79 (79.8%)	201 (83.1%)	
*Comorbidity*				
HTN	2 (2.2%)	0 (0.0%)	2 (2.0%)	1.000
HLP	1 (1.1%)	0 (0.0%)	1 (1.0%)	
Others	17 (19.1%)	2 (18.2%)	19 (19.0%)	
> 1	7 (7.9%)	1 (9.1%)	8 (8.0%)	
*Pathologic Stage of disease*				
I&II	6 (9.1%)	15 (16.9%)	21 (13.5%)	0.235
III	60 (90.9%)	74 (83.1%)	134 (86.5%)	
*EUS Stage of disease*				
I&II	7 (18.4%)	2 (40.0%)	9 (20.9%)	0.277
III	31 (81.6%)	3 (60.0%)	34 (79.1%)	
*Location of tumor*				
Upper	1 (1.4%)	0 (0.0%)	1 (0.6%)	< 0.001
Middle	60 (81.1%)	13 (14.9%)	73 (45.3%)	
Lower	13 (17.6%)	74 (85.1%)	87 (54.0%)	

The patients’ medical records were reviewed for age, sex, chief complaint, preoperative comorbidities, disease stage, surgical procedure, postoperative course, and short-term survival. An upper endoscopy was performed and all patients had biopsy-proven esophageal squamous cell carcinoma. Disease staging was determined by chest and abdomen CT scan with or without endoscopic ultrasonography (EUS). In order to evaluate the surgical risk assessment, lung and heart consultation were done. Although today in our medical centers, neoadjuvant chemo radiotherapy is recommended in all esophageal cancer with stage higher than T1N0, 91 (37.45%) of the studied populations received adjuvant chemo-radiation. The selection of the operative technique (TTE or THE) is based primarily on the tumor location, surgeon’s experience and institution policy. The selected procedure was performed by or under supervision of an experienced investigator surgeon. In the THE technique, using midline incision, the esophagus and adjacent lymph nodes were dissected through diaphragm hiatus and thoracic inlet. Then the created gastric conduit was pulled up through the posterior mediastinum, and cervical anastomosis was performed. Transthoracic esophagectomy consisted of right posterolateral thoracotomy for esophagectomy and mediastinal lymph node dissection. Through laparotomy, for gastric conduit preparation, cervical anastomosis was performed similar to transhiatal procedure. Pyloromyotomy was conducted in all patients of the groups.

The main measures for outcome assessment were postoperative complications, mortality and survival. The collected data were analyzed using descriptive statistics such as frequency distribution for categorical variables, and mean and standard deviation for numerical variables. Qualitative data were analyzed by using chi-square and Fisher’s exact tests in cross tabulation.

## Results

Among 243 patients enrolled in the study, 126 (51.9%) were female and the remaining 117 (48.1%) were male. The age ranged between 18 and 85 years (16.9% between 18 and 50, 83.1% between 50 and 85). In 87 (54.0%) patients, the tumor was originated from the lower thoracic esophagus, in 73 (45.4%) patients, cancer was located in the middle thoracic esophagus, the tumor was observed in the upper thoracic esophagus of one (0.6%) patient, and the data of others were missed. In 147 (61.8%) patients, the medical team ordered preoperative chemo-radiation and 91 patients received adjuvant chemo-radiation only. The surgical technique was THE (the Orringer method) in 99 (40.7%) and TTE (the McKeown method) in 144 (59.3%) patients. Except for sex, patient demographic data for two groups were not statistically different (Table 1).

Of the patients, 234 (96.3%) patients suffered from dysphagia as the main chief complaint which statistically showed no significant difference between the groups (*p* = 0.213).

The most common site of tumor in the TTE group was the middle esophagus (60, 81.1%) and the lower esophagus in 74 (85.1%) cases of the THE group which revealed a statistically significant difference between groups (*p* < 0.001). Fisher's Exact Test revealed no significant association between preoperative comorbidities and surgery type (*p* = 1.000). According to CT scan and EUS findings, most of the patients had stage III disease and no significant difference was observed between groups (*p* = 0.277). Finally, according to histologic TNM classification, 21 (13.5%) patients were in stage I and II, 134 (86.5%) patients were in stage III and the values of others were missing. Based on Fisher’s exact test, there was no significant difference concerning the stage in each surgical technique group (*p* = 0.235).

The mean follow up time was 17.6 ± 15.0 months. Postoperative complications occurred in 94 patients whose data are presented in Table 2. The most common complication was recurrence of dysphagia within three months after surgery (22, 9.1%) which was more common in the THE group, but there was not any significant difference between the groups (*p* = 0.152). No difference was also found between the groups in terms of pulmonary complications, such as aspiration pneumonia and respiratory failure (*p* = 0.559). Three patients in the TTE group and one patient in the THE group developed transient hoarseness from injury to recurrent laryngeal nerve with no significant difference (*p* = 0.159). Three (2.1%) patients in two groups developed anastomotic leakage (There were two (2.3%) patients in the THE, and one (1.8%) patient in the TTE group and the difference was not significant, *p* = 0.854). Four patients in the TTE group and two patients in the THE group developed delayed gastric emptying which revealed no significant difference (*p* = 0.204). The operative mortality was considered as any death occurred during the same hospitalization or within first 30 days after the operation, which amounted to 5.3%. The operative mortality was seen in six (10.0%) patients in the TTE group and two (2.2%) patients in the THE group (*p* = 0.035).

**Table 2:** Post-operative complications among two studied group

Group	TTE	THE	Total	*p*-value
*Delayed gastric emptying*				
No	51 (92.7%)	86 (97.7%)	137 (95.8%)	0.204
Yes	4 (7.3%)	2 (2.3%)	6 (4.2%)	
*Dysphagia relapse during 3 months*				
No	50 (90.9%)	71 (80.7%)	121 (84.6%)	0.152
Yes	5 (9.1%)	17 (19.3%)	22 (15.4%)	
*Tracheal injury*				
No	54 (98.2%)	88 (100.0%)	142 (99.3%)	0.385
Yes	1 (1.8%)	0 (0.0%)	1 (0.7%)	
*Chylothorax*				
No	54 (98.2%)	86 (97.7%)	140 (97.9%)	1.000
Yes	1 (1.8%)	2 (2.3%)	3 (2.1%)	
*Respiratory complication*				
No	53 (96.4%)	87 (98.9%)	140 (97.9%)	0.559
Yes	2 (3.6%)	1 (1.1%)	3 (2.1%)	
*Anastomosis stricture*				
No	55 (100.0%)	87 (98.9%)	142 (99.3%)	1.000
Yes	0 (0.0%)	1 (1.1%)	1 (0.7%)	
*Recurrent nerve palsy*				
No	52 (94.5%)	87 (98.9%)	139 (97.2%)	0.159
Yes	3 (5.5%)	1 (1.1%)	4 (2.8%)	
*Cardiac problems*				
No	55 (100.0%)	85 (96.6%)	140 (97.9%)	0.285
Yes	0 (0.0%)	3 (3.4%)	3 (2.1%)	
*Mortality of first 30 days after surgery*				
No	54 (90%)	87 (97.8%)	141 (94.7%)	*p* = 0.035
Yes	6 (10%)	2 (2.2%)	8 (5.3%)	
*Anastomosis leakage*				
No	54 (98.2%)	86 (97.7%)	140 (97.9%)	1.000
Yes	1 (1.8%)	2 (2.3%)	3 (2.1%)	
*Total*				
No	18 (32.7%)	31 (35.2%)	49 (34.3%)	0.759
Yes	37 (67.3%)	57 (64.8%)	94 (65.7%)	

## Discussion

The ultimate goal in esophageal cancer surgery is achieving the best oncological results with the least morbidity and mortality to improve patients’ quality of life. But there is still controversy about the best surgical approach. Based on the previous studies, transthoracic esophagectomy offers better visualization of the tumor and a more extended lymph node resection with better long-term oncologic outcomes, whereas transhiatal esophagectomy has limited perioperative morbidity and mortality [8–10]. In present investigation on 243 patients with esophageal SCC, demographic features of the patients were similar to those of previous published studies. Most of the patients (83.1%) were above 50 years old with no significant difference between the TTE and THE groups. But, a statistically significant difference was found in the number of women compared with men underwent the surgery. The frequency distribution of patients’ comorbidities was similar in both groups. Although in previous studies both procedures exhibited similar survival, in some studies the THE technique has been acknowledged to decrease surgical risk which is better tolerated by patients [11, 12]. In our study, the overall incidence of patients having at least one postoperative complication was 65.7%, but the incidence of each complication did not differ significantly between the groups.

The results of this study showed that the TTE technique was associated with higher incidence of delayed gastric emptying, tracheal and recurrent nerve palsy injury, and respiratory complication. Higher incidence of delayed gastric emptying may be because in this procedure the stomach is floating in the thoracic cavity without any surrounding compression. The higher incidence of tracheal and recurrent nerve palsy injury in this group can be attributable to the proximity of trachea and the nerve in the upper and middle part of the esophagus, and given that in our study the majority of tumors were located in the middle esophagus. Also, impaired pulmonary toilet duo to the pain, preoperative poor pulmonary function and performance status, and notably surgical approach could be the possible causes for higher incidence of respiratory complication in this group. On the other hand, the THE group had a higher incidence of dysphagia relapse, chylothorax, anastomosis stricture and fistula. However, there was no significant difference between two groups. The THE procedure was associated with decreased incidence of chylothorax, which can be ascribed to better exposure and ligation of the thoracic duct during surgery. These findings are to some extent comparable with the results of other similar studies [13, 14]. Also, cardiovascular problems and other minor complications including pleural effusion were comparable between two groups.

In a meta-analysis of 52 studies, Boshier et al. showed that with similar 5-year survival, the TTE group had significantly more respiratory complications, wound infections, and early postoperative mortality, while anastomotic leak or stricture and recurrent laryngeal nerve injury rate were significantly higher in THE group [15]. In a randomized controlled trial for comparing TTE and THE, Hulscher et al. indicated that although THE was associated with fewer pulmonary complications and shorter length of hospital stay, the overall disease-free and quality-adjusted survival did not differ statistically between groups [9].

In a similar randomized trial, Omloo et al. reported that the 5-year survival was not significantly different in the THE and TTE groups, but patients with a limited number of positive lymph nodes in resected specimen may benefit from transthoracic esophagectomy [10]. Vocal cord paralysis due to recurrent laryngeal nerve injury during cervical anastomoses can be seen after both transthoracic and transhiatal procedures, but it is a transient complication that resolves within a few months [16, 17]. In multiple studies, the rate of recurrent nerve palsy was reported 9–14% in the TTE and 7–11% in the THE group [18–20]

In our study, transient hoarseness was reported in 2.8% of patients and two groups had similar results owing to the same procedure in performing cervical anastomosis. This complication was resolved in postoperative follow up and none of the patients required intubation or tracheostomy. In our opinion, thanks to not using metal retractors in the neck and gentle dissection at the tracheoesophageal groove, the incidence of recurrent laryngeal nerve injury was lower in our patients.

Studies reported higher rate of anastomosis leakage, without any difference in both groups [8, 21, 22]. In our series, the incidence of leakage was 2.1%, which was not different in two groups. High volume surgery in our centers may be an important factor for this lower incidence. It appears that early identification, appropriate neck, thoracic and mediastinal drainage, and sometimes, timely reoperation are of most importance for handling this complication. In our study, with the same age distribution and the same risk factors, the in-hospital and 30-day mortality rate was 5.3% for all patients (more common in the TTE group with a significant difference; *p* = 0.035) which was lower than mortality rates in large database studies [14, 23]. Lymph node metastasis is one of the most important prognostic factors in esophageal cancer and the chance of cure among patients is highly dependent on lymph node involvement and extent of lymphadenectomy. Siewert and Stein suggested that extensive lymphadenectomy in TTE surgery can improve the prognosis in patients with early stage disease but concerning advanced lymphatic metastases, this method can only result in the reduction of local recurrences. Extensive lymphadenectomy in TTE may increase the morbidity but has better prognosis in proximal tumors and limited involved lymph nodes [24]. In our study, we did not find any associations between these two treatment groups in terms of lymphadenectomy outcome, which it might be due to the short follow-up duration.

The advancement in surgical techniques and improved perioperative care of patients have led to significant increases in survival, however, the results of multiple studies have shown that in the hands of experienced surgeons there is no superiority between two procedures in short term in terms of oncological outcomes [1, 6, 25, 26]. Another study showed that although TTE and THE esophagectomy had similar complication rates, operative mortality, and 90-day re-admission rates, the THE technique was associated with lower costs and shorter length of hospital stay [22]. Other studies reported resembling outcomes after THE and TTE [27].

Because of the retrospective nature of our study, the main limitation was missing data such as perioperative transfusion, operative time, number of retrieved lymph nodes and reoperations regarding the complexity of the procedure. Also, there were no data available fulfillment of pain assessment test and pain management. Comorbidities like COPD and smoking condition were not defined clearly and despite routine use of the nasogastric tube and chest tube, no data were available about the timing of their removal. Unfortunately, because of the absence of EUS in the past, tumor characteristics influencing the choice of procedure were unknown, which might have led to a selection bias. According to the hospitals policy, for large or high located tumors or bulky lymph node metastasis, a transthoracic operation was planned which was associated with increased morbidity than lower stage tumors.

## Conclusion

Despite the improvements in preoperative risk assessment, operative techniques and postoperative intensive care management, esophageal cancer surgery is still associated with a high incidence of operative complications and high mortality rate. We concluded that the decision about the best kind of surgical technique for esophageal cancer treatment can be made by considering patients’ co-morbidities, cancer pathology and stage, location of tumor and surgeons’ experience. Longer follow up is required to elucidate which procedure has an advantage in disease control and long-term survival.

## Data Availability

Not applicable.

## References

[1] Enzinger PC, Mayer RJ (2003). Esophageal cancer. N Engl J Med.

[2] Delpisheh A, Veisani Y, Sayehmiri K, Rahim E (2014). Esophageal carcinoma: long-term survival in consecutive series of patients through a retrospective cohort study. Gastroenterol Hepatol Bed Bench.

[3] Yarhusseini A, Sharifzadeh L, Delpisheh A, Veisani Y, Sayehmiri F, Sayehmiri K (2014). Survival Rate of Esophageal Carcinoma in Iran—A Systematic Review and Meta-analysis. Iran J Cancer Prev.

[4] Koca T, Arslan D, Basaran H, Cerkesli AK, Tastekin D, Sezen D, Koca O, Binici DN, Bassorgun CI, Ozdogan M (2015). Dietary and demographical risk factors for oesophageal squamous cell carcinoma in the Eastern Anatolian region of Turkey where upper gastrointestinal cancers are endemic. Asian Pac J Cancer Prev.

[5] Chen MF, Yang YH, Lai CH, Chen PC, Chen WC (2013). Outcome of patients with esophageal cancer: a nationwide analysis. Ann Surg Oncol.

[6] Stilidi I, Davydov M, Bokhyan V, Suleymanov E (2003). Subtotalesophagectomy with extended 2-field lymph node dissection for thoracic esophageal cancer. Eur J Cardiothorac Surg.

[7] Manzoni GD, Pedrazzani C, Pasini F, Di Leo A, Durante E, Castaldini G, Cordiano C (2002). Results of surgical treatment of adenocarcinoma of the gastric cardia. Ann Thorac Surg.

[8] Patti MG (2010). Transhiatal versus transthoracic esophagectomy for esophageal cancer. World J Gastroenterol.

[9] Hulscher JB, van Sandick JW, de Boer AG, Wijnhoven BP, Tijssen JG, Fockens P, Stalmeier PF, ten Kate FJ, van Dekken H, Obertop H, Tilanus HW, van Lanschot JJ (2002). Extended transthoracic resection compared with limited transhiatal resection for adenocarcinoma of the esophagus. N Engl J Med.

[10] Omloo JM, Lagarde SM, Hulscher JB, Reitsma JB, Fockens P, van Dekken H, Ten Kate FJ, Obertop H, Tilanus HW (2007). Extended transthoracic resection compared with limited transhiatal resection for adenocarcinoma of the mid/distal esophagus: five-year survival of a randomized clinical trial. Ann Surg.

[11] Orringer MB, Marshall B, Stirling MC (1993). Transhiatal esophagectomy for benign and malignant disease. J Thorac Cardiovasc Surg.

[12] Boyle MJ, Franceschi D, Livingstone AS (1999). Transhiatal versus transthoracic esophagectomy: complication and survival rates. Am Surg.

[13] Rindani R, Martin CJ, Cox MR (1999). Transhiatal versus Ivor-Lewis oesophagectomy: Is there a difference?. Aust N Z J Surg.

[14] Hulscher JB, Tijssen JG, Obertop H, van Lanschot JJ (2001). Transthoracic versus transhiatal resection for carcinoma of the esophagus: a meta-analysis. Ann Thorac Surg.

[15] Boshier PR, Anderson O, Hanna GB (2011). Transthoracic versus transhiatal esophagectomy for the treatment of esophagogastric cancer: a meta-analysis. Ann Surg.

[16] Scholtemeijer MG, Seesing MFJ, Brenkman HJF, Janssen LM, van Hillegersberg R, Ruurda JP (2017). Recurrent laryngeal nerve injury after esophagectomy for esophageal cancer: incidence, management, and impact on short- and long-term outcomes. J Thorac Dis.

[17] Koyanagi K, Igaki H, Iwabu J, Ochiai H, Tachimori Y (2015). Recurrent laryngeal nerve paralysis after esophagectomy: respiratory complications and role of nerve reconstruction. Tohoku J Exp Med.

[18] Altorki N, Kent M, Ferrara C, Port J (2002). Three-field lymph node dissection for squamous cell and adenocarcinoma of the esophagus. Ann Surg.

[19] Swanson SJ, Batirel HF, Bueno R, Jaklitsch MT, Lukanich JM, Allred E, Mentzer SJ, Sugarbaker DJ (2001). Transthoracic esophagectomy with radical mediastinal and abdominal lymph node dissection and cervical esophago gastrostomy for esophageal carcinoma. Ann Thorac Surg.

[20] Orringer MB, Marshall B, Iannettoni MD (1999). Transhiatal esophagectomy: clinical experience and refinements. Ann Surg.

[21] Wei MT, Zhang YC, Deng XB, Yang TH, He YZ, Wang ZQ (2014). Transthoracic vs transhiatal surgery for cancer of the esophagogastric junction: a meta-analysis. World J Gastroenterol.

[22] Khullar OV, Jiang R, Force SD, Pickens A, Sancheti MS, Ward K, Gillespie T, Fernandez FG (2015). Transthoracic versus transhiatal resection for esophageal adenocarcinoma of the lower esophagus: a value-based comparison. J Surg Oncol.

[23] Rentz J, Bull D, Harpole D, Bailey S, Neumayer L, Pappas T, Krasnicka B, Henderson W, Daley J, Khuri S (2003). Transthoracic versus transhiatal esophagectomy: a prospective study of 945 patients. J Thorac Cardiovasc Surg.

[24] Siewert JR, Stein HJ (1999). Lymph-node dissection in squamous cell esophageal cancer—who benefits?. Langenbecks Arch Surg.

[25] Donohoe CL, O’Farrell NJ, Ravi N, Reynolds JV (2012). Evidence-based selective application of transhiatal esophagectomy in a high-volume esophageal center. World J Surg.

[26] Łochowski M, Łochowska B, Kozak J (2017). Transthoracic versus transhiatal esophagectomy—influence on patient survival. Gastroenterol Rev.

[27] Connors RC, Reuben BC, Neumayer LA, Bull DA (2007). Comparing outcomes after transthoracic and transhiatal esophagectomy: a 5-year prospective cohort of 17,395 patients. J Am Coll Surg.

